# Astragaloside IV, as a potential anticancer agent

**DOI:** 10.3389/fphar.2023.1065505

**Published:** 2023-02-17

**Authors:** Dongqin Xia, Wenjie Li, Ce Tang, Juan Jiang

**Affiliations:** ^1^ Chongqing University Cancer Hospital, Chongqing, China; ^2^ Affiliated Hospital of Northwest Minzu University, Lanzhou, China; ^3^ State Key Laboratory of Southwestern Chinese Medicine Resources, School of Ethnic Medicine, Chengdu University of Traditional Chinese Medicine, Chengdu, China

**Keywords:** astragaloside IV, AS-IV, natural product, anticancer, saponin

## Abstract

Cancer is a global intractable disease, and its morbidity and mortality are increasing year by year in developing countries. Surgery and chemotherapy are often used to treat cancer, but they result in unsatisfactory outcomes, such as severe side effects and drug resistance. With the accelerated modernization of traditional Chinese medicine (TCM), an increasing body of evidence has shown that several TCM components have significant anticancer activities. Astragaloside IV (AS-IV) is considered the main active ingredient of the dried root of *Astragalus membranaceus*. AS-IV exhibits various pharmacological effects, such as anti-inflammatory, hypoglycemic, antifibrotic, and anticancer activities. AS-IV possesses a wide range of activities, such as the modulation of reactive oxygen species-scavenging enzyme activities, participation in cell cycle arrest, induction of apoptosis and autophagy, and suppression of cancer cell proliferation, invasiveness, and metastasis. These effects are involved in the inhibition of different malignant tumors, such as lung, liver, breast, and gastric cancers. This article reviews the bioavailability, anticancer activity, and mechanism of AS-IV and provides suggestions for further research of this TCM.

## Introduction

Cancer, the leading cause of death globally, is not only a major public health problem but also an important barrier to improving life expectancy (Sung et al., 2021). According to the 2019 data from World Health Organization, cancer is the first or second leading cause of death in 183 countries and third or fourth in other regions (World Health Organization, 2020). Approximately 19.3 million new cancer cases and 10 million cancer deaths worldwide were predicted in 2020, and 28.4 million new cancer cases worldwide are expected in 2040, an increase of 47% from the 2020 data (Singh et al., 2022). The global cancer burden is currently growing at an alarming rate, but no particular effective treatments that can curb the spread of cancer are currently available (Sun et al., 2017).

Cancer treatment often depends on the type of tumor, stage of diagnosis, and the patient’s underlying condition. Available cancer treatment options include surgical excision, chemotherapy, radiotherapy, hormone therapy, and targeted therapy (DeSantis et al., 2014). Given the advances in early diagnosis and treatment of cancer, the number of cancer patient survivors is increasing annually (Singh et al., 2022). However, based on clinical factors related to treatment, several side effects, such as postoperative tumor spread or metastasis and chemotherapy resistance, can significantly affect patient prognosis (Sun et al., 2017). Therefore, studies should aim at searching for candidate agents that can selectively induce cancer cell death without affecting normal cells and increase the sensitivity to chemotherapy drugs.

Traditional Chinese medicine (TCM) is becoming increasingly recognized worldwide because of its low toxicity, low side effects, and good tolerance. TCM plays an indispensable role in cancer prevention and treatment by preventing tumor occurrence, reducing toxicity, enhancing therapeutic effects (e.g., radiotherapy and chemotherapy), and reducing tumor recurrence and metastasis. Astragali Radix is mainly derived from the dried roots of the leguminous plant *A. membranaceus* (Fish.) Bge. var. Mongholicus (Bge.) Hsiao or/and *A. membranaceus* (Fish.) Bge. (Chinese Pharmacopoeia Commission., 2020). This TCM is classified as a tonic in *Shennong’s Herbal Classic* and *Compendium of Materia Medica* and can improve body immunity. Astragali Radix contains various chemical components, such as saponins, flavonoids, and polysaccharides (Zheng et al., 2020). In Chinese pharmacopoeia, astragaloside IV (AS-IV) is used as the quality control index. AS-IV is the main active substance of Astragali Radix, which possesses anticardiovascular disease (Wang Q. N. et al., 2021), liver protection (Liang et al., 2021), antidiabetic nephropathy (Xing et al., 2021), and antitumor activities (Xu et al., 2018). AS-IV exerts antitumor effects on various cancer models, such as lung (Li et al., 2021a), liver (Jiang and Mao, 2019), and colorectal (Ye et al., 2017) carcinomas. In addition, AS-IV can be used in combination with other antitumor drugs and increase the sensitivity of chemotherapy drugs (Zheng et al., 2018). AS-IV is also non-toxic and presents good safety.

This study was performed to review the anticancer activity and mechanism of action of AS-IV in different cancers, such as lung, liver, colorectal, breast, gastric, and cervical cancers and glioma. The cancer preventive effects of AS-IV were systematically summarized.

## Understanding of cancer in traditional Chinese medicine

Experts of TCM have had a certain understanding of tumor and cancer a long time ago. The discussion about tumor and cancer appeared in the classics, such as *The Yellow Emperor’s Inner Canon* and *The Classic of Medical Problems*, more than 2000 years ago (Liu et al., 2015; Guo et al., 2022). TCM names cancers differently than Western medicine. Mammary cancer resembles breast cancer, and abdominal mass, hepatic accumulation, and stony goiter belong to ovarian, liver, and thyroid cancers, respectively (Zhu and Wen, 2018). In TCM, cancer occurrence is believed to be mainly due to the deficiency in healthy *Qi* caused by the imbalance of *Yin* and *Yang*, the perception of evil poison, emotional disorder, and diet injury, which lead to the dysfunction of viscera and the abnormal operation of *Qi*, blood, and body fluid; such condition produces pathological changes, including *Qi* stagnation, blood stasis, phlegm, heat toxicity, dampness, and turbidity. These pathological changes are located in the viscera, which struggle with the body and accumulate over time to form a malignant disease (Zhang et al., 2007; Chen and Shen, 2022).

The overall treatment of cancer In TCM mainly involves strengthening the body resistance and eliminating evil, including nourishing *Qi* and *blood*, warming *Yang*, and nourishing *Yin*, supplemented by activating blood and resolving stasis, softening hardness to dissipate stagnation, clearing heat, and detoxification (Xie et al., 2017; Li et al., 2021b; Wang et al., 2022a). The TCM treatment of cancer emphasizes the role of strengthening the body resistance and eliminating evil, which means that it strengthens body resistance to maintain the stability of the immune system and eliminates evil by directly eradicating cancer cells (Zhu and Wen, 2018). Astragali Radix invigorates *Qi* for consolidation of the exterior (Yang et al., 2019a), induces diuresis to alleviate edema, and promotes pus discharge and tissue regeneration (Dong et al., 2021); its characteristics of strengthening body resistance and eliminating evil are especially suitable for cancer prevention and treatment.

## Clinical application of *Astragalus membranaceus* in cancer

In TCM, Astragali Radix is mainly used in the clinical treatment of cancer in the form of prescription formulation and compatibility, such as the use of *Huangqi Guizhi Wuwu* decoction for blood vessel growth after cancer operation and peripheral neurotoxicity caused by chemotherapeutic drugs (Huo and Jia, 2022). In modern preparations, Astragali Radix exists in the form of astragalus injection, and quality control limits the dosage of AS-IV to no less than 0.08 mg/mL. Astragalus injection combined with chemotherapy can prevent leukopenia caused by chemotherapy, protect normal cells, improve clinical symptoms of malignant tumor patients after chemotherapy, and reduce adverse reactions (Wang and Feng, 2009). Astragalus injection at Zusanli acupoint can improve the quality of life and behavior of patients with advanced tumors (Meng et al., 2009).

## Chemical properties of AS-IV

**FIGURE 1:**
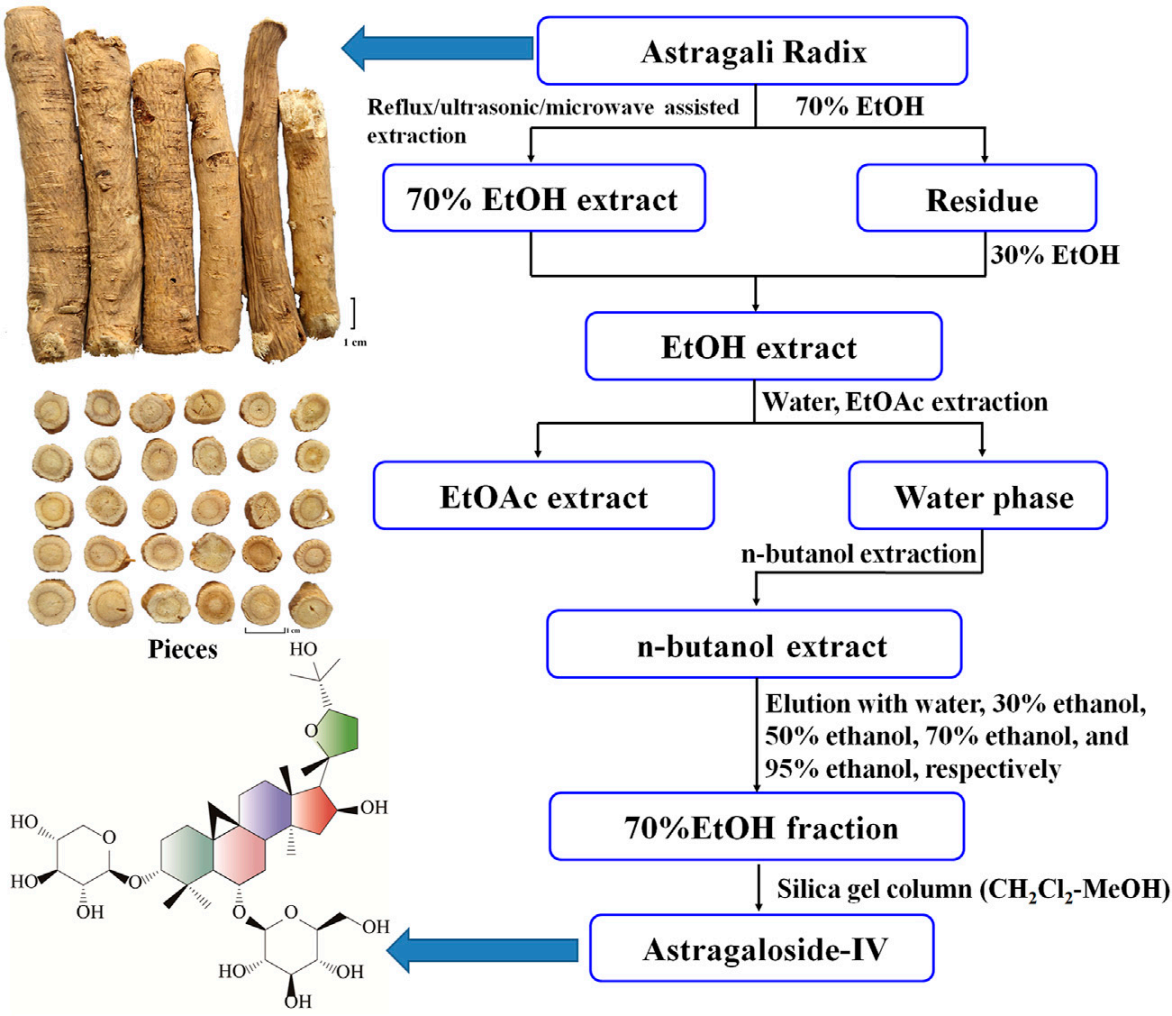
Flow chart for isolating AS-IV from Astragali Radix.

AS-IV is also known as astrasieversianin XIV, astraversianin XIV, or cyclosiversioside F. AS-IV is a white powder with a melting point of 284°C–285°C (Wang et al., 1987). Its molecular formula is C_41_H_68_O_14_, and its theoretically accurate molecular weight is 784.4609 (Kitagawa et al., 1983). AS-IV is a glycoside component and an oligosaccharide of xylose and glucose. The aglycone obtained by AS-IV hydrolysis is cycloastragenol, and the link between xylose and glucose is at C_3_ and C_6_, respectively. Thus, this molecule is named 3-*O*-*β*-D-xylopyranosyl-6-*O*-*β*-glucopyranosyl-cycloastragenol. Figure 1 shows the structural formula (Kitagawa et al., 1983). AS-IV is a secondary metabolite produced during plant metabolism. Figure 1 also demonstrates the general process of AS-IV separation from plants. In Chinese Pharmacopoeia, AS-IV is mainly used as the main quality control index for Astragali Radix. Given that AS-IV is a saponin component with a weak ultraviolet absorption, evaporative light-scattering detector, charged aerosol detector, or mass spectrometry is often used to determine its content. The 2020 edition of Chinese Pharmacopoeia stipulates that the content of AS-IV in Astragali Radix should not be less than 0.080% (Chinese Pharmacopoeia Commission, 2020).

## Anticancer effects of AS-IV

The main anticancer effects of AS-IV include cell cycle arrest in the G_0_/G_1_ phase (Jiang and Mao, 2019), induction of apoptosis (Liu et al., 2020; Guo et al., 2021) through the stimulation of endoplasmic reticulum (ER) stress and mitochondrial-dependent apoptosis pathway, induction of autophagy (Xia et al., 2020), and inhibition of cell proliferation, invasion, and metastasis (Zhang et al., 2019). In addition, AS-IV can inhibit tumor growth in tumor model mice (Xia et al., 2020) and enhance the chemical sensitivity to chemotherapy drugs, such as cisplatin (He et al., 2016). These effects were limited to tumor cells and did not induce cytotoxic effects in normal cells. AS-IV can also exert effects on lung (Li et al., 2021a), liver cancer (Qu et al., 2020), colorectal cancer (Wang et al., 2018a), breast cancer (Hu et al., 2020), glioma (Han et al., 2020), gastric cancer (Liu et al., 2021), cervical cancer (Xia et al., 2020), prostate cancer (He et al., 2021), ovarian cancer (Wang et al., 2021b), abdominal aortic aneurysm (Wang J. N. et al., 2018), osteosarcoma (Hu et al., 2017), and vulvar cancer (Zhao et al., 2019) (Table). Therefore, AS-IV is a promising anticancer agent with favorable pharmacological effects.

### Molecular targets

**FIGURE 2:**
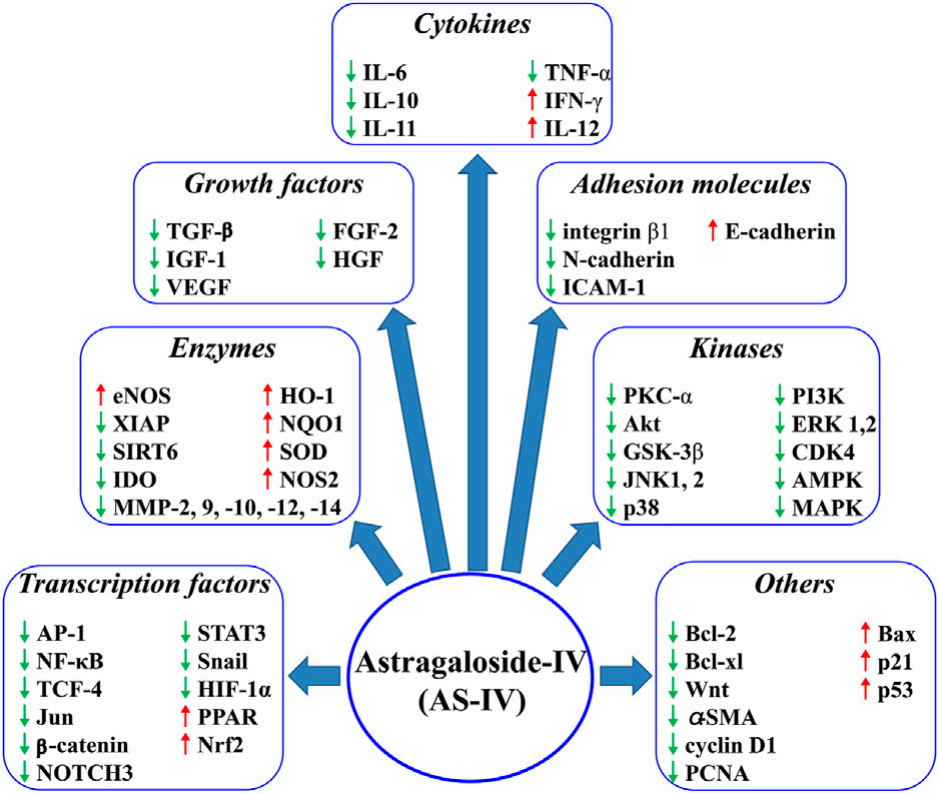
AS-IV regulates several molecular targets.

AS-IV can exert anticancer effects through various pathways: inhibition of pro-inflammatory agents, such as cytokines, by nuclear factor (NF)-κB; alteration of several growth factors expression, including vascular endothelial growth factor (VEGF), transforming growth factor (TGF)-β, and hepatocyte growth factor (HGF); consumption of adhesion molecules (intercellular adhesion molecule-1); regulation of several cell survival or cell cycle genes, such as cyclin D1, p21, and Bcl-2; regulation of kinases, including mitogen-activated protein kinase (MAPK), AMP-activated protein kinase (AMPK), and phosphatidylinositol-3 kinase (PI3K); activation of antioxidant reaction by Nrf2 (Figure 2).

### Inhibition of proliferation and induction of cell cycle arrest

Tumor growth is closely related to the infinite proliferation of cancer cells (Tian et al., 2020). Cell proliferation is regulated by DNA replication and cell cycling-related proteins, such as proliferating cell nuclear antigens (PCNA), Ki67, cyclin dependent kinases (CDKs), and p21 (Karakas et al., 2006; Yang et al., 2017). AS-IV can dose-dependently inhibit p21 expression in colorectal cancer cells, which leads to cell accumulation in the G0 phase, and inhibit PCNA expression (Sun et al., 2019). In addition, AS-IV (50 and 100 ng/mL) can significantly decrease the levels of cyclin D1 and CDK4 in SW620 cells (Wang et al., 2018a). Cyclin D1 is a well-characterized target gene of NF-κB, which can bind to the promoter of cyclin D1 to stimulate its transcription. NF-κB promotes cancer cell proliferation by regulating cyclin D1 and bypassing G1 cell cycle checkpoints. AS-IV inactivates NF-κB by reducing the phosphorylation level of p65. B7-H3 is a regulator of the NF-κB pathway. Thus, the anticancer effect of AS-IV may be dose-dependently (50 and 100 ng/mL) realized by the underlying B7-H3/NF-κB/cyclin D1 axis (Wang et al., 2018a).

### Induction of apoptosis

Apoptosis, also known as programmed cell death, is beneficial to normal cell development, organ growth, and homeostasis of tissues (Rogers and Alnemri, 2019). Apoptosis is a normal physiological process, and it plays an important role in the development and homeostasis of organisms. Defects in apoptosis occur in most cancers, such as lung, breast, liver, prostate, and bladder cancers (Tang et al., 2020). From the perspective of mechanism, apoptosis can be activated by intrinsic mitochondrial and extrinsic death receptor apoptosis pathways. When the mitochondrial apoptosis pathway is activated, cells directly or indirectly perceive intracellular or extracellular stimuli, such as DNA damage, reactive oxygen species, and hypoxia (Banoth and Cassel, 2018). These stimuli ultimately disrupt mitochondrial function by inducing the expression and activation of pro-apoptotic members of the Bcl-2 family, such as Bcl-2, Bcl-xL, and Bak (Huang et al., 2019). By contrast, stimulated extrinsic death receptors induce continuous activation of caspase-3, which cleaves target proteins and leads to apoptosis (Ke et al., 2021).

Table 1 shows that AS-IV mainly induces apoptosis through the above intrinsic mitochondrial and extrinsic death receptor apoptosis pathways. In terms of the intrinsic mitochondrial pathway, AS-IV can activate caspase-3/-7/-9 by promoting the release of cytochrome c (cyt c) from mitochondria. In addition, Bcl-2 can inhibit the release of cyt c and avoid the intrinsic mitochondrial apoptosis induced by Bax. A large number of studies have shown that AS-IV can reduce the level of Bcl-2, promote the expression of Bax, and increase the ratio of Bax/Bcl-2, including that observed in lung cancer, non-small-cell lung cancer, and liver, colorectal, breast, and vulvar cancers. AS-IV administration in the range of 200–800 μg/mL upregulated Bax and cleaved caspase 3 expression, suppressed Bcl2 and Bcl-xL levels, and increased the mortality of vulvar squamous cancer cells (SW962) (Zhao et al., 2019).

**TABLE 1:** Protective effect and mechanism of AS-IV anticancer.

Cancer type	Cell type	Observation	Effect	Mechanism	Reference
Lung cancer	A549	*In vitro* (5, 10, 20 *μM*)	↓migration	↓MMP-2, ↓MMP-9, ↓integrin β1, ↓TGF-β1	Cheng et al. (2014)
			↓invasion	↓TNF-α, ↓IL-6, ↓PKC-α	
			↓inflammation	↓p-ERK1/2, ↓NF-κB (p65)	
				↑E-cadherin	
Lung cancer	A549 H1299	*In vitro* (80, 160 μg*/*mL)	↓migration	↓p-AMPKα, ↓CCL7, ↓MMP9, ↓MMP10, ↓MMP14, ↓VEGFA	Xu et al. (2018)
		*In vivo* male C57BL/6 J mice of 5 weeks old (40 mg/kg)	↓invasion	↓ICAM-1, ↓IGF-1, ↓CCL2	
			↓angiogenesis		
Lung cancer	A549	*In vitro* (10, 20, 50, 100 ng*/*mL)	↑autophagy	↑P62, ↓Beclin1, ↓CTSB	Li et al. (2021a)
			↑chemosensitivity	↓CTSL, ↑cleaved caspase 3, ↓Bcl-2, ↑Bax, ↑p-AKT	
			↑apoptosis	↑p-mTOR	
Non-small cell lung cancer	A549	*In vitro* (10, 20, 40 ng*/*mL)	↑chemosensitivity	↓B7-H3	He et al. (2016)
	HCC827 NCI-H1299				
Non-small cell lung cancer	NCI-H1299	*In vitro* (3, 6, 12, 24 ng*/*mL)	↓proliferation	↓SIRT6	Dai et al. (2017)
	HCC827		↑chemosensitivity		
	A549				
Non-small cell lung cancer	HCC827	*In vitro* (12, 24 ng*/*mL)	↓proliferation	↓Bcl-2, ↑Bax, ↓p-Akt	Jia et al. (2019)
	A549 NCI-H1299		↓migration	↓p-GSK-3β, ↓p-β-catenin	
			↑apoptosis	↑cleaved caspase 3	
Non-small cell lung cancer	A549 NCI-H1299	*In vitro* (8, 16 ng*/*mL)	↑chemosensitivity	↓GRP78, ↓Beclin1	Lai et al. (2020)
			↓endoplasmic reticulum stress		
			↑autophagy		
Liver cancer	Bel-7402	*In vitro* (0.08, 0.16 mg*/*mL)	↑drug resistance	↓P-gp, ↓MDR1	Wang et al. (2014)
			↑intracellular accumulation		
Liver cancer	HepG2	*In vivo* male BALB/c nude mice of 5–6 weeks old (20 mg/kg)	↓tumor growth	↓VEGF, ↓FGF-2, ↓MMP-2	Zhang et al. (2017)
			↓tumor vascularization	↓HGF, ↓TF, ↓FVII	
				↑miR-122, ↓miR-221	
Liver cancer	Huh7	*In vitro* (10, 50, 100 μg*/*mL)	↓migration	↑E-cadherin, ↓N-cadherin	Qin et al. (2017)
	MHCC97-H		↓invasion	↓p-AKT, ↓p-GSK-3β	
				↓β-catenin	
Liver cancer	Bel-7402	*In vitro* (0.1 *mM*)	↑drug resistance	↓p-JNK, ↓p-c-Jun, ↓P-gp	Wang et al. (2017)
				↓AP-1	
Liver cancer	SK-Hep1	*In vitro* (200, 400 *μM*)	↓viability	↑cleavage-caspase-3/8/9, ↓XIAP, ↓MCL1, ↓C- FLIP	Su et al. (2020)
			↓proliferation trigger G1 arrest	↓survivin	
	Hep3B		↑apoptosis		
			↓invasion		
Liver cancer	HepG2	*In vitro* (40 *μM*)	↑chemosensitivity	↓MRP2	Qu et al. (2020)
		*In vivo* H22 male BALB/c nude mice of 4 weeks old (50 mg/kg)	↓Cis-induced kidney injury		
Liver cancer	HepG2	*In vitro* (25, 50, 100 *nM*)	↑G2/M arrest	↑cleaved-caspase-3/9	Jiang and Mao (2019)
		*In vivo* HepG2 male BALB/c nude mice (50 mg/kg)	↑G0/G1 arrest	↑E-cadherin, ↓N-cadherin	
			↓proliferation	↓vimentin, ↓Wnt	
	Hep3B		↑apoptosis	↓β-catenin, ↓TCF-4	
			↓invasion	↓VEGF, ↓MMP-14	
			↓migration		
Liver cancer	SMMC-7721	*In vitro* (160 μg*/*mL)	↓migration	↓lncRNA-ATB	Li et al. (2018)
	Huh7		↓viability	↑E-cadherin, ↓N-cadherin	
			↓epithelial-mesenchymal transition (EMT)	↓IL-11, ↓p-STAT3	
Liver cancer	HepG2	*In vitro* (0.8 μg*/*mL)	↓viability	↓PI3K, ↓p-PI3K, ↓AKT	Guo et al. (2021)
			↓migration	↓p-AKT, ↓mTOR	
			↓invasion	↓p-mTOR, ↑GNGT1	
			↓cell cycle		
			↑apoptosis		
			↓proliferation		
Liver cancer	HSC-T6	*In vitro* (5, 10, 20 *μM*)	↓liver injury	↑p-Smad3C, ↑p-Nrf2, ↑HO-1, ↑NQO1	Zhang et al. (2021)
		*In vivo* male C57BL/6J mice of 6 weeks old (20, 40, 80 mg/kg)	↓fibrosis	↓p-Smad2C, ↓p-Smad2L	
				↓p-Smad3L, ↓PAI-1	
	HepG2			↓ α-SMA, ↓ TGF-β1	
				↓ALT, ↓AST, ↑SOD	
				↓ MDA, ↓AFP, ↓c-Myc	
Colorectal cancer	SW620	*In vitro* (50, 100 ng*/*mL)	↓proliferation	↓cyclin D1, ↓CDK4	Wang et al. (2018a)
	HCT116		↓cell cycle	↓B7-H3, ↓p-P65 NF-κB	
				↓miR-29c	
Colorectal cancer	SW480	*In vitro* (5, 10, 25, 50 μg*/*mL)	↑chemosensitivity	↑miR-134, ↓CREB1	Ye et al. (2017)
			↓proliferation	↑E-cadherin, ↓N-cadherin	
			↓migration	↓Snail, ↓Vimentin	
			↓invasion		
Colorectal cancer	HCT116	*In vitro* (100 ng*/*mL)	↓viability	↓NOTCH3	Xie et al. (2016)
	SW480		↑chemosensitivity		
Colorectal cancer	CT26	*In vitro* (100 *nM*)	↓growth of tumor	↓Arg1, ↓Mrc1,↑NOS2	Liu et al. (2020)
		*In vivo* CT26 tumor-bearing Balb/c mice (15 mg/kg)	↑apoptosis	↓TGF-β, ↓IL-10, ↑IL-12,↓VEGF-A, ↑IFN-γ, ↑TNF-α	
			↓proliferation		
Colorectal cancer	HT29	*In vitro* (10, 20,40 μg*/*ml)	↓proliferation	↑p21, ↑Bax/Bcl-2, ↑C0079 t C	Sun et al. (2019)
	SW480	*In vivo* athymic BALB/c mice 20 mg/kg)	↑cell cycle arrest	↑Omi, ↑PARP, ↓PCNA	
			↑apoptosis	↑cleaved caspase-3/9	
Breast cancer	MCF-7	*In vitro* (20, 40,80 μg*/*mL)	↓proliferation	↑TRHDE-AS1, ↓MMP-2	Hu et al. (2020)
	MDA-MB-231	*In vivo* MDA-MB-231 BALB/c nude mice (20 mg/kg)	↓migration	↓MMP-9, ↓PCNA	
Breast cancer	MCF-7	*In vitro* (30, 50 *μM*)	↑chemosensitivity	↓CAV-1, ↑3-NT	Zheng et al. (2018)
	MDA-MB-231	*In vivo* MDA-MB-231female Balb/c nude mice (50 mg/kg)	↑apoptosis	↓p-ERK1/2, ↑eNOS	
				↑NO	
Breast cancer	MDA-MB-231	*In vitro* (10, 20,40 μg*/*mL)	↓viability	↓Vav3, ↓p-ERK1/2	Jiang et al. (2017)
		*In vivo* MDA-MB-231female athymic Balb/c nude mice (20 mg/kg)	↓invasion	↓p-JNK, ↓MMP-2, ↓MMP-9	
			↓migration		
			↓proliferation		
Glioma	U251	*In vitro* (20, 40,80 μg*/*mL)	↓migration	↑E-cadherin, ↓N-cadherin	Han et al. (2020)
			↓invasion	↓vimentin, ↓β-catenin	
			↑apoptosis	↓cyclin-D1	
			↓proliferation		
Glioma	U251	*In vitro* (40, 60,80 μg*/*mL)	↓proliferation	↓Ki67, ↓PCNA, ↓MMP-2	Li et al. (2017)
		*In vivo* U251Athymic BALB/c mice (20 mg/kg)	↓migration	↓MMP-9, ↓VEGF, ↓C-myc	
			↓invasion	↓p-ERK1/2, ↓p-MAPK	
Gastric cancer	SGC7901	*In vitro* (10 μg*/*mL)	↑EMT	↑E-cadherin, ↓N-cadherin	Liu et al. (2021)
	MGC803		↓angiogenesis	↓Snail, ↓vimentin, ↓VEGF	
			↓proliferation		
Gastric cancer		*In vivo* MNNG male Sprague–Dawley rat (50, 100 mg/kg)	↑gastric mucosa	↓LDHA, ↓CD147, ↓MCT1	Zhang et al. (2018)
			↓glycolysis process	↓MCT4, ↓HIF-1α, ↑TIGAR	
				↑miRNA-34a, ↑p53	
Gastric cancer	BGC-823	*In vitro* (10, 20 μg*/*mL)	↓viability	↓N-cadherin, ↓Vimentin	Zhu et al. (2018)
			↑EMT	↑E-cadherin, ↓MMP-2/9	
	MKN-74		↓invasion	↓Snail, ↓p-Akt, ↓p-p65	
			↓migration		
Cervical cancer	HeLa	*In vitro* (5, 10, 25 *μM*)	↓proliferation	↑LC3I/II, ↑Atg7, ↑Atg12	Xia et al. (2020)
		*In vivo* SiHa BALB/c nude mice (12.5, 25, 50 mg/kg)	↓invasion	↑DCP1A, ↑TMSB4X	
	SiHa		↓xenograft tumor growth		
			↑autophagy		
Cervical cancer	SiHa	*In vitro* (50, 200, 800 μg*/*mL)	↓proliferation	↓N-cadherin, ↓Vimentin	Zhang et al. (2019)
		*In vivo* SiHa BABLc/nude mice	↓invasion	↑E-cadherin, ↓Vimentin	
			↓migration	↓TGF-β1, ↓p-P38, ↓p-PI3K	
			↓EMT	↓p-ERK1/2, ↓p-JNK1/2	
				↓p-mTOR	
Prostate cancer	LNCap	*In vitro* (10 *μM*)	↑chemosensitivity	↓p-AKT, ↓p-p65, ↑p-IκBα	He et al. (2021)
	PC-3	*In vivo* PC-3 BABLc/nude mice (40 mg/kg)	↑apoptosis	↑Cleaved PARP	
Ovarian cancer	THP-1	*In vitro* (10 μg*/*mL)	↓proliferation	↓PCNA, ↓HMGB1, ↓IL-10	Wang et al. (2021b)
			↓migration	↓TLR4, ↓TGF-β, ↓MMP-9	
Vulvar cancer	SW962	*In vitro* (200, 400, 600, 800 μg*/*mL)	↓proliferation	↑P53, ↑P21, ↓cyclin D1, ↑Bax, ↑cleaved-caspase-3	Zhao et al. (2019)
			↑G0/G1 phase arrest	↓Bcl-xl, ↓Bcl-2, ↑Beclin-1	
			↑apoptosis	↑LC3-B, ↓P62, ↑TGF-βRII	
			↑autophagy	↑Smad4	
Abdominal aortic aneurysm	RAW264.7	*In vitro* (2, 10, 50 μg*/*mL)	↓inflammation	↓NF-κB, ↓CCL-1, ↓TNF-α	Wang J. N. et al. (2018)
		*In vivo* Bap/Ang II male C57/B6j mice (20, 80 mg/kg)	↓oxidative stress	↓ROS, ↓MMP-9/12, ↓p-P65	
				↓p-IκB, ↑p-AKT	
Osteosarcoma	MG-63	*In vitro* (40 *μM*)	↓proliferation	↑cleaved-caspase-3/8, ↑cleaved-PARP, ↑Fas, ↑FasL	Hu et al. (2017)
	143B	*In vivo* 143B cells BALB/c nu/nu mice (20 mg/kg)	↑chemosensitivity		
			↑apoptosis		

↑: upregulates; ↓: downregulates; alpha fetoprotein (AFP); protein kinase B (Akt); AMP-activated protein kinase (AMPK); activator protein-1 (AP-1); autophagy related (Atg); GPI-linked cd59 and costimulatory molecule cd276 (B7-H3); Bcl-2-associated X protein (Bax); B-cell lymphoma 2 (Bcl-2); caveolin-1 (CAV-1); C-C motif chemokine (CCL); cyclin-dependent kinase (CDK); cAMP-response element binding protein (CREB); cathepsin B (CTSB); CTD, small phosphatase-like protein (CTSL); cytochrome c (Cyt C); extracellular regulated protein kinases (ERK); endothelial growth factor (FGF); cellular FLIcE-like inhibitory protein (c-FLIP); coagulation factor VII (FVII); G Protein Subunit Gamma Transducin 1 (GNGT1); glucose regulated protein 78 (GRP78); glycogen synthase kinase 3β (GSK-3β); hypoxia-inducible factor-1 (HIF-1α); hepatocyte growth factor (HGF); Heme oxygenase-1 (HO-1); intercellular adhesion molecule 1 (ICAM-1); insulin-like growth factor I (IGF-1); IκB kinase α (IκBα); interleukin-10 (IL-6); c-Jun N-terminal kinase (JNK); Transcription factor Jun (Jun); the protein expressions of light chain 3I/II (LC3I/II); lactate dehydrogenase (LDHA); mitogen-activated protein kinase (MAPK); myeloid-cell-leukemia 1 (MCL1); monocarboxylate transporter (MCT); multidrug resistance protein 1 (MDR1); matrix metalloproteinase (MMP); multidrug resistance-associated protein 2 (MRP2); mammalian target of rapamycin (mTOR); nuclear factor-κB (NF-κB); Nitric Oxide Synthase 2 (NOS2); NAD(P)H: quinone oxidoreductase 1 (NQO1); Nuclear factor erythroid 2-related factor 2 (Nrf2); 3-nitrotirosina (3-NT); plasminogen activator inhibitor 1 (PAI-1); poly AdP-ribose polymera (PARP); proliferating cell nuclear antigen (PCNA); P-glycoprotein (P-gp); phosphoinositide-3-kinase (PI3K); protein kinase c system -α (PKC-α); signal transducer and activator of transcription 3 Rac1, Rac family small GTPase, 1 (STAT3); NAD-dependent protein deacetylase sirtuin-6 (SIRT6); alpha smooth muscle actin (α-SMA); superoxide dismutase (SOD); Transcription factor 4 (TCF-4); tissue factor (TF); transforming growth factor-β1 (TGF-β1); TP53-induced glycolysis and apoptosis regulatorPLGC(TIGAR); tumor necrosis factor α (TNF-α); vascular endothelial growth factor (VEGF); X-linked inhibitor of apoptosis protein (XIAP).

In terms of extrinsic death receptor apoptosis, several receptors, such as Fas ligands and tumor necrosis factor-α (TNF-α), can initiate caspase-8-dependent external apoptosis pathways, which are activated after caspase cascade reaction, and finally trigger apoptosis (Zhou et al., 2021). Fas is an important death receptor that can mediate FasL to induce apoptosis (Laubach et al., 2019). FasL plays a key role in the inhibition of tumor development. During *in vitro* (dose 40 µM) and *in vivo* (dose 20 mg/kg) studies of osteosarcoma, AS-IV up-regulated the Fas/FasL-triggering caspase cascade, which resulted in the activation of caspase-3/8. The final activated caspase-3 cleaved poly ADP ribose polymerase and triggered apoptosis (Hu et al., 2017).

Prevention of the abnormal progression of apoptosis is an important reason for tumor development. Overexpressed anti-apoptotic proteins (XIAP, MCL1, C-FLIP, and survivin) can weaken the therapeutic effect of anticancer drugs by blocking the apoptosis mediated by internal and external pathways (Kim et al., 2021). AS-IV can reverse the overexpression of anti-apoptotic proteins. AS-IV at concentrations of 200–400 µM can significantly inhibit the expressions of anti-apoptotic proteins (XIAP, MCL1, C-FLIP, and survivin) and induce the apoptosis of SK-Hep 1 and Hep 3B cells (Su et al., 2020).

### Inhibition of the cancer progression and metastasis

Epithelial–mesenchymal transition (EMT) plays an important role in early tumor invasion and metastasis; it is the process of transformation from early-onset to malignant tumors (Pastushenko and Blanpain, 2019). EMT causes tumor cells to lose their epithelioid phenotype, obtain more mesenchymal cyto-like characteristics, reduce intercellular adhesion, and gain invasive ability. AKT is generally considered a cancer gene, and it is overexpressed in numerous types of solid tumors, involved in various basic cellular processes, and closely associated with EMT in cancer (Yu et al., 2019). The activation of AKT leads to the loss of junctions between tumor cells, disruption of tumor cell polarity and morphological changes, and enhancement of tumor cell motility (Xu et al., 2015). Matrix metalloproteinases are metastasis-related genes involved in EMT. Snail is an important EMT-related transcription factor that affects metastasis-related genes (Lee et al., 2017). In addition, several proteins, such as E-cadherin, N-cadherin, vimentin, α-smooth muscle actin (SMA), and Slug, are closely related to EMT (Phillips and Kuperwasser, 2014; Odero-Marah et al., 2018). Previous studies have shown that AS-IV inhibited the migration and invasion of hepatocellular carcinoma cells in a dose-dependent manner. AS-IV can up-regulate the expression of E-cadherin and down-regulate those of N-cadherin, vimentin, α-SMA, and Slug. Notably, AS-IV treatment significantly reduced the phosphorylated forms of AKT and glycogen synthase kinase (GSK)-3β, which in turn inhibited the expression of β-catenin. Therefore, AS-IV at dose of 10–100 μg/mL can inhibit EMT by targeting the AKT/GSK-3β/β-catenin pathway, thus weakening the invasion and migration of Huh7 and MHCC97-H cells (Qin et al., 2017). AS-IV (dose 10 and 20 μg/mL) can also inhibit tumor metastasis by inhibiting TGF-β1 and inducing EMT to inhibit the PI3K/AKT/NF-κB pathway in BGC-823 and MKN-74 cells (Zhu et al., 2018).

Vav protein is the guanosine nucleotide exchange factor of Rho family GTP enzymes, in which Vav3 is a proto-oncogene whose carcinogenic activity is mediated by different downstream pathways, including PI3K and MAPK pathways (Bustelo, 2014). Studies have shown that Vav3 can be involved in breast and prostate cancers by activating estrogen and androgen receptors, respectively (Bar-Shavit et al., 2016; Culig, 2016). In addition, Vav3 promotes the invasion and migration of glioblastoma cells and neuroblasts and plays a role in the invasion, growth, and metastasis of oral squamous cell carcinoma. Jiang et al. (2017) observed that AS-IV inhibits the proliferation and invasion of breast cancer cells *in vitro* and inhibits tumor growth by down-regulating Vav3 and Rac1/MAPK *in vivo* (dose 50 mg/kg). In addition, AS-IV at dose of 50–800 μg/mL can reduce the invasion and migration ability of cervical cancer cells (SiHa) by inhibiting pP38 and PI3K, which down-regulates the expression of TGF-β1 (Zhang et al., 2019).

MAPK/extracellular signal-regulated kinase (ERK) signaling pathway can regulate cell proliferation, apoptosis, and invasion by phosphorylating a variety of substrates; thus, it plays a key role in the occurrence and development of a variety of tumors (Guo et al., 2020). AS-IV significantly inhibited MAPK/ERK signal transduction in glioma cells (dose 40–80 μg/mL) and tumor-bearing mice (20 mg/kg), which showed decreases in the levels of p-MEK, p-ERK, and C-myc (Li et al., 2017). Long non-coding RNA (lncRNAs) are a kind of transcripts with no protein coding potential, and numerous lncRNAs play key roles in the occurrence and development of cancer (Huang et al., 2021). LncRNAs activated by TGF-β (lncRNA-ATB) promote EMT and metastasis by competitive binding of miR-200 and the survival of cancer cells by activating interleukin (IL)-11/signal transducer and activator of transcription 3 (STAT3) signal pathway. LncRNA-ATB and its downstream targets and biological processes, including EMT, migration, IL-11/STAT3 signal transduction, and hepatoma cell survival, are all regulated by AS-IV (Li et al., 2018).

The protein kinase C (PKC)/ERK1/2 pathway plays an important role in the survival, proliferation, apoptosis, migration, and invasion of cancer cells (Isakov, 2017; Roskoski, 2019). The overexpression of PKC is considered one of the biomarkers for cancer diagnosis, and it can be activated by phorbol esters and promote the development of tumor. PKC mediates tumor cell migration and invasion through downstream signal pathways, such as ERK1/2 (Cheng et al., 2014). Thus, inhibiting the expression of isomer PKC-α can inhibit tumor cell invasion and migration. Cheng et al. (2014) reported that AS-IV at the dose of 10 μM inhibited the migration and invasion of A549 cells by regulating the PKC-α-ERK1/2-NF-κB signal pathway.

M2 polarized macrophages are commonly called tumor-associated macrophages (TAMs) (Choi et al., 2018). They promote the growth, invasion, metastasis, and angiogenesis of cancer cells and are one of the main tumor-infiltrating immune cells (Boutilier and Elsawa, 2021). Clinical studies and experimental evidence show that M2 macrophages are responsible for promoting tumor activity, including tumor-related angiogenesis; tumor initiation, progression, and metastasis; intravascular injection; inhibition of anti-tumor immune response (Jayasingam et al., 2020; Boutilier and Elsawa, 2021). Xu et al. (2018) discovered that AS-IV significantly inhibited the invasion, migration, and angiogenesis of A549 and H1299 cells induced by M2-CM. In addition, *in vivo* (dose 40 mg/kg) experiments showed that AS-IV can significantly inhibit the growth of Lewis lung cancer and reduce metastasis. AS-IV can also inhibit the activation of AMPKα in M2 macrophages, and silencing of AMPKα can partially block the inhibitory effect of AS-IV. These results suggest that AS-IV can inhibit the progression and metastasis of lung cancer by regulating macrophage polarization through the AMPK signal.

### Inhibition of angiogenesis

Abnormal angiogenesis is considered the hallmark of malignant tumors (Viallard and Larrivée, 2017). An increasing amount of clinical evidence indicates that angiogenesis is closely related to metastasis in the prognosis of cancer surgery (García-Figueiras et al., 2015). The formation of new blood vessels promotes the development of tumor, and angiogenesis is a dynamic process involving several key factors with angiogenic activity. VEGF is the most relevant factor in clinics. It can stimulate endothelial cell proliferation and induce neovascularization (Pulkkinen et al., 2021). Fibroblast growth factor 2 (FGF-2) easily binds to FGF receptor and leads to angiogenesis (Eguchi and Wakabayashi, 2020). In addition, HGF is a kind of stromal cytokine that can promote tumor angiogenesis by stimulating vascular endothelial cell migration and activating protein kinase B and ERK (Shabbir et al., 2015). Programmed death-ligand 1 (PD-L1) is not only a stimulating factor of tumor-associated fibroblasts, but it can also promote the growth and angiogenesis of tumor cells (Gulley et al., 2022). Several studies have shown that TF and FVII promote tumor angiogenesis by initiating exogenous coagulation pathways (Kasthuri et al., 2009; Kocatürk and Versteeg, 2013). AS-IV can reverse these situations. Liu et al. (2021) revealed that AS-IV (10 μg/mL) inhibited angiogenesis in gastric cancer cells (SGC7901 and MGC803) by regulating microRNA-195-5p-mediated PD-L1. AS-IV can also significantly reduce the expressions of VEGF, FGF2, HGF, TF, and FVII and inhibit the growth and angiogenesis of orthotopic transplanted tumor in nude mice (Zhang et al., 2017).

### Enhancement of chemosensitivity

In the treatment of malignant tumors, the resistance of the body to chemotherapeutic drugs is the main reason for treatment failure (Bukowski et al., 2020). With the increased use of chemotherapeutic drugs, drug resistance has become a great challenge. AS-IV can increase the sensitivity of tumor chemotherapeutic drugs. Several researchs demonstrated that chemotherapy resistance is mainly mediated by P-glycoprotein (P-gp) (Jiang et al., 2021; Shah et al., 2023). Inhibition of P-gp transporters and regulation of multidrug resistance (MDR) are important strategies for reversing MDR (Pilotto Heming et al., 2022). Wang et al. (2017) reported that AS-IV at the dose of 0.1 mM can down-regulate the expression of MDR1 by inhibiting the c-Jun N-terminal kinase/c-Jun/activator protein 1 signaling pathway, which reverses the drug resistance of Bel-7402/FU cells.

MDR protein 2 (MRP2) is an ATP-binding cassette transporter and contributes to the MDR of tumor cells. It can regulate the outflow of chemotherapeutic drugs from tumor cells to reduce drug concentration in tumor cells (Chen et al., 2015). Continuous-exposure and antineoplastic drugs can induce the overexpression of MRP2 in tumor cells, which reduces the accumulation of intracellular drugs (Chen et al., 2015). Therefore, the overexpression of MRP2 is one of the important mechanisms to reduce chemosensitivity and cause MDR. Qu et al. (2020) observed that oral administration of 50 mg/kg AS-IV can inhibit the overexpression of MRP2 in tumor tissues of BALB/c nude mice bearing H22 tumor and enhance the chemosensitivity of hepatoma cells to cisplatin.

B7-H3, a member of B7 family, plays a key role in carcinogenesis and tumor progression (Flem-Karlsen et al., 2019). Up-regulation of B7-H3 exists in a variety of cancers, such as lung cancer, colorectal cancer, pancreatic cancer (Carvajal-Hausdorf et al., 2019; Ma et al., 2020; Wang et al., 2022b). The mRNA and protein levels of B7-H3 in non-small-cell lung cancer cells treated with AS-IV and cisplatin decreased significantly, which indicated that AS-IV (dose 5 ng/mL) significantly down-regulated the expression of B7-H3 and increased the sensitivity to cisplatin (He et al., 2016).

Sirtuin 6 (SIRT6) is a kind of NAD^+^-dependent III deacetylase, and it can regulate the occurrence of cancer, including liver and breast cancers, by regulating a variety of cellular signal pathways (Hu et al., 2018; Song et al., 2020). In addition, SIRT6 is considered a potential prognostic indicator and a therapeutic target for chemosensitivity prediction (Hu et al., 2018). AS-IV at the dose of 3–6 ng/mL can promote the sensitivity of gefitinib by up-regulating SIRT6 in NSCLC cells (NCI-HI299, HCC827, and A548) (Dai et al., 2017).

ER is an essential organelle in eukaryotic cells and has a variety of functions. The change in tumor cell microloops or the action of antitumor drugs can trigger ER stress-activated unfolded protein response, which increases the level of ER molecular chaperones, such as GRP78, and ER stress-sensitive protein PERK (Lei et al., 2021). Lai et al. (2020) stated that cisplatin can trigger ER stress and increase the chemotherapy resistance of cancer cells. The combination of AS-IV and cisplatin enhances the sensitivity of cancer cells to cisplatin and the anti-tumor effect of cisplatin through ER stress.

Caveolin-1 (CAV-1) is an important constituent protein of special membrane depression called fossa and a potential target for the prevention of cancer drug resistance and improvement of the clinical prognosis of various kinds of malignant tumors (Chung et al., 2015). With regard to the potential mechanism, an increasing number of evidence showed that CAV-1 is closely related to the redox signal of cancer cells. Zheng et al. (2018) confirmed that AS-IV at the dose of 30 μM enhanced the chemosensitivity of breast cancer to paclitaxel by inhibiting the activation of eNOS/NO/3-NT signal pathway by CAV-1 in MCF-7 and MDA-MB-231 cells.

### Promotion of atutophagy

Autophagy is a process in which proteins or organelles are swallowed into vesicles and fused with lysosomes to induce autophagy (Onorati et al., 2018). The wrapped contents are degraded, which meets the metabolic needs of cells and renewal of certain organelles. Autophagy plays a two-way regulatory role in tumor development. Autophagy can affect the expression of p62, autophagy component protein Lc3-I/II, and autophagy-related protein Beclin-1 (Chiu et al., 2022). AS-IV (dose 50 ng/mL) promotes the expression of p62 and Lc3-I/II and inhibits the expressions of Beclin-1 and lysosomal CTSB and CTSL to inhibit autophagy in A549 cells (Li et al, 2021a). In addition, AS-IV induced autophagy significantly increases the expression of autophagy-associated proteins, namely, Bcelin-1 and Lc3-I/II, in a dose-dependent manner (8 and 16 ng/mL) to enhance the anti-tumor effect and chemotherapy resistance of cisplatin in A549 and H1299 cells (Lai et al., 2020). Meanwhile, AS-IV (dose 25 μM) can significantly increase the expressions of Atg7 and Atg12 in HeLa and SiHA cells (Xia et al., 2020). In VSCC SW962 cells, AS-IV (800 μg/mL) significantly increased the levels of Beclin-1 and Lc3-II and decreased the expression level of p62 (Zhao et al., 2019). AS-IV inhibited the expressions of lysosome CTSB and CTSL by triggering autophagy to enhance the sensitivity of lung adenocarcinoma cells to bevacizumab (Li et al, 2021a).

### Inhibition of inflammation

The occurrence and development of tumors are closely related to the microenvironment of inflammation (Iyengar et al., 2016). A large number of inflammatory cells, especially macrophages, exist before tumor invasion. Tumor-associated macrophages regulate inflammation and adaptive immunity by producing TGF-β1 and cytokines (TNF-α and IL-6) and promote angiogenesis and cell proliferation (Zhukova et al., 2022). The expression of TNF-α in the tumor microenvironment is a common feature of numerous malignant tumors, and it can promote the metastasis and invasion of various kinds of tumor cells (Wu and Zhou, 2010). Inflammatory cytokine IL-6 is another important inflammatory cytokine, and it is closely related to inflammation and tumor. IL-6 can activate the TGF-β1 pathway to promote cancer cell invasion (Zhukova et al., 2022). Macrophages in the tumor microenvironment regulate inflammation and adaptive immunity by producing growth factors (e.g., TGF-β1) and cytokines (e.g., TNF-α and IL-6), thereby enhancing angiogenesis (Hinshaw and Shevde, 2019). Cheng et al. (2014) used enzyme-linked immunosorbent assay to detect the levels of inflammatory factors in the supernatant fluid of A549 cells. They observed that AS-IV at the dose of 5–20 μM significantly reduced the levels of TGF-β1, TNF-α, and IL-6 and inhibited the activation of NF-κB. AMPK is involved in the polarization of M2 macrophages, and AS-IV can induce a sharp decrease in p-AMPK levels. These results suggest that AS-IV can inhibit the activation of AMPKα in M2 macrophages. *In vivo* studies have also demonstrated that AS-IV (40 mg/kg) can significantly inhibit tumor growth and reduce the number of metastases in Lewis lung cancer (Xu et al., 2018).

**FIGURE 3:**
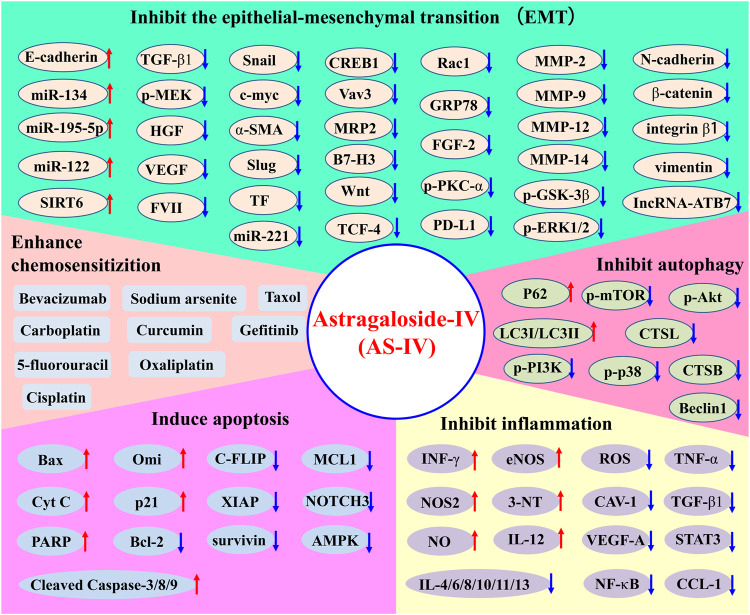
Overview of the anticancer pharmacological activities of AS-IV.

In conclusion, AS-IV has significant efficacy in various cancers and has been studied to varying degrees *in vivo* and *in vitro*. Figure 3 and Table 1 summarize the anticancer pharmacological activities of AS-IV.

## Pharmackinetics of AS-IV

Pharmacokinetics refers the description of drug absorption, distribution, metabolism, and excretion. In-depth understanding of drug pharmacokinetics can be used to assess the properties and application prospects of drugs. In 2004, Gu et al. (2004) analyzed administration for the first time the pharmacokinetics of AS-IV in rat plasma after oral using liquid chromatography-mass spectrometry technology. Du et al. (2005) investigated the changes in absorption after the oral administration of AS-IV to rats. When 20 mg/kg AS-IV was orally administered, the mean time to peak concentration (T_max_), half-life (T_1/2_), mean residence time (MRT), and clearance (CL) were 0.75, 3.8, and 4.62 h and 6.16 L kg^-1^·h^-1^, respectively. The absolute bioavailability of oral administration of AS-IV was 3.66%. Zhang et al. (2007) used beagle dogs to investigate the pharmacokinetics of AS-IV and observed that after oral administration, T_max_, T_1/2_, MRT, and CL were 1.0, 3.83, and 4.35 h and 0.010 L kg^-1^·min^-1^, respectively. In addition, AS-IV was widely distributed in diverse tissues in the body, with the greatest distribution in the liver and lungs, and subsequently rapidly eliminated from most tissues (Zhang et al., 2006).

**FIGURE 4:**
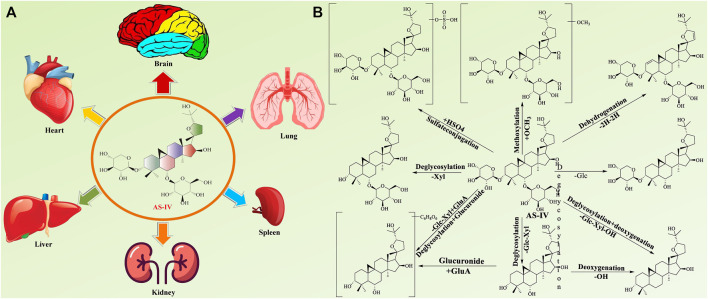
*In vivo* tissue distribution **(A)** and major metabolites of AS-IV **(B)**.

The above studies showed that AS-IV has a low bioavailability and high absorption and elimination rates. In TCM, Astragali Radix is often used together with other TCMs, such as Puerarin Radix and Atractylodis macrocephalae Rhizoma. Puerarin can significantly increase the plasma peak concentration of AS-IV and reduce the oral clearance rate. These characteristics suggest that puerarin can significantly change the pharmacokinetic characteristics of AS-IV by increasing the absorption of AS-IV or inhibiting its metabolism *in vivo* (Zhang et al., 2020). Other TCM components, such as triptolide (Gao et al., 2020), cycloastragenol (He et al., 2018), atractylenolide I (Song et al., 2014), and prim-O-glucosylcimifugin (Song et al., 2014), can also change the pharmacokinetic characteristics of AS-IV. Drugs play an important role in the identification of metabolites *in vivo*, and a systematic study of AS-IV metabolism *in vivo* can aid in determining its pharmacological mechanism. Cheng and Wei. (2014) analyzed the metabolites of AS-IV in the plasma, bile, and stool samples of rats. They identified 22 metabolites, mainly including parent chemicals and phases I and II metabolites. Metabolic reactions primarily comprised hydrolysis, glucuronidation, sulfation, and dehydrogenation (Figure 4).

## Toxicity of AS-IV

*In vitro* cell experiments have shown that AS-IV, at concentrations up to 400 μg/mL, had no effect on RAW264.7 cell viability (Ying et al., 2021). In addition, AS-IV (0.1–100 µM) had no significant effect on the viability of normal cultured H9c2 cells (Yang et al., 2019b). Low concentrations of AS-IV (3 and 6 ng/mL) did not significantly change the cell viability of NCIH1299, HCC827, and A549 cells. However, high doses of AS-IV (12 and 24 ng/mL) significantly inhibited their proliferation (Dai et al., 2017). A large number of animal studies have confirmed that AS-IV is not toxic at all stages of animal growth, except during pregnancy and perinatal period. Zhu et al. (2009) discovered fetal toxicity at intravenous doses higher than 0.5 mg/kg and maternal toxicity in rats at doses higher than 1.0 mg/kg. However, no teratogenic effects were found in rats and rabbits. These results suggest that AS-IV is safe for most cells at low concentrations and toxic for cancer cells at relatively high concentrations. Although other researchs have reported that AS-IV had certain effects on pregnant animals, such relevant studies are limited. Whether this finding is an accidental experimental result needs to be further studied. Thus, AS-IV is a relatively safe chemical component.

## Conclusion and future perspectives

The aforementioned research advancements support the anticancer potential of AS-IV. This TCM inhibits carcinogenesis in all parts of the body. To date, numerous findings have highlighted the role of AS-IV in ongoing EMT, with EMT playing a role in most processes associated with AS-IV in cancer. However, most of the studies were performed using *in vitro* cell experiments. The dose and efficacy of AS-IV to cancer cells are not comparable to those of the human body because the body’s immune system can regulate the degradation of various enzymes. Several studies on mouse animal models have also validated the anticancer effects of AS-IV, but the results have been limited given the small number of research. In addition, AS-IV has been demonstrated to sensitize or improve drug resistance during antitumor chemotherapy.

Combined with the results of this review and compared with the development of other TCM, to date, no study has reported the clinical research on AS-IV, and only information about *Astragalus* extract injection is available. The use of AS-IV may be limited in these areas. First, Astragali Radix has the main role of replenishing *qi* in the traditional use of TCM, and the effect of replenishing *qi* is inferior to that of Ginseng. Numerous ginseng studies have been conducted, but those on Astragali Radix are relatively limited, where Astragali Radix was mainly used as an auxiliary drug for diabetes and compared with other major hypoglycemic drugs, such as insulin injection and metformin, that have been invested in proprietary medicine development again. Second, although several studies have shown that AS-IV combined with other chemotherapeutic drugs can improve chemosensitivity, the mechanism is still unclear. In addition, AS-IV violates Lipinski’s “Rule of Five,” and its bioavailability in animals is low and unstable. Although this manuscript only reviewed the application of AS-IV in cancer, a large number of studies have shown that AS-IV has various functions, such as improving immunity, assisting hypoglycemia, assisting anti-cancer, improving chemotherapy sensitivity. In view of the above three limitations, after recognizing the irreplaceability of AS-IV, we can increase the investment in AS-IV and conduct in-depth studies to clarify the mechanism of AS-IV’s broad-spectrum anticancer, determine adjuvant anticancer proper, and enhance chemosensitivity. In addition, further clinical studies are needed to confirm its multiple effects. Finally, the low bioavailability and instability of AS-IV are solved by the modification of its chemical structure or the targeting of modern nano-delivery systems. In conclusion, AS-IV has a great potential as a neglected broad-spectrum anticancer drug and an effective adjuvant drug for cancer treatment.
